# Conduction system pacing versus conventional pacing in patients undergoing atrioventricular node ablation: Nonrandomized, on-treatment comparison

**DOI:** 10.1016/j.hroo.2022.04.005

**Published:** 2022-05-04

**Authors:** Pugazhendhi Vijayaraman, Andrew J. Mathew, Angela Naperkowski, Wilson Young, Parash Pokharel, Syeda A. Batul, Randle Storm, Jess W. Oren, Faiz A. Subzposh

**Affiliations:** ∗ Geisinger Heart Institute, Wilkes Barre, Pennsylvania; † Geisinger Heart Institute, Scranton, Pennsylvania; ‡ Geisinger Heart Institute, Danville, Pennsylvania

**Keywords:** AV node ablation, Conduction system pacing, His bundle pacing, Left bundle branch area pacing, Right ventricular pacing, Biventricular pacing, Death, Heart failure hospitalization

## Abstract

**Background:**

Atrioventricular node ablation (AVNA) with right ventricular or biventricular pacing (conventional pacing; CP) is an effective therapy for patients with refractory atrial fibrillation (AF). Conduction system pacing (CSP) using His bundle pacing or left bundle branch area pacing preserves ventricular synchrony.

**Objective:**

The aim of our study is to compare the clinical outcomes between CP and CSP in patients undergoing AVNA.

**Methods:**

Patients undergoing AVNA at Geisinger Health System between January 2015 and October 2020 were included in this retrospective observational study. CP or CSP was performed at the operators’ discretion. Procedural, pacing parameters, and echocardiographic data were assessed. Primary outcome was the combined endpoint of time to death or heart failure hospitalization (HFH) and was analyzed using Cox proportional hazards. Secondary outcomes were individual outcomes of time to death and HFH.

**Results:**

AVNA was performed in 223 patients (CSP, 110; CP, 113). Age was 75 ± 10 years, male 52%, hypertension 67%, diabetes 25%, coronary disease 40%, and left ventricular ejection fraction (LVEF) 43% ± 15%. QRS duration increased from 103 ± 30 ms to 124 ± 20 ms (*P* < .01) in CSP and 119 ± 32 ms to 162 ± 24 ms in CP (*P* < .001). During a mean follow-up of 27 ± 19 months, LVEF significantly increased from 46.5% ± 14.2% to 51.9% ± 11.2% (*P* = .02) in CSP and 36.4% ± 16.1% to 39.5% ± 16% (*P* = .04) in CP. The primary combined endpoint of time to death or HFH was significantly reduced in CSP compared to CP (48% vs 62%; hazard ratio 0.61, 95% confidence interval 0.42–0.89, *P* < .01). There was no reduction in the individual secondary outcomes of time to death and HFH in the CSP group compared to CP.

**Conclusion:**

CSP is a safe and effective option for pacing in patients with AF undergoing AVNA in high-volume centers.

## Graphical abstract

**Figure:**
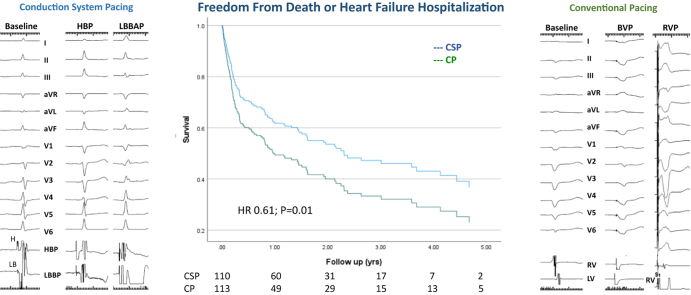

**Key Findings**

▪ Conduction system pacing (CSP) using His bundle pacing and left bundle branch area pacing was feasible and safe in patients undergoing atrioventricular (AV) node ablation.
▪ Electrical and procedural outcomes of CSP were comparable to conventional pacing (CP) using right ventricular pacing and biventricular pacing in patients undergoing AV node ablation.
▪ CSP may be associated with improvement in the combined outcome of time to death or heart failure hospitalization compared to CP, although this was likely owing to a sicker group of patients with lower baseline left ventricular ejection fraction and wider QRS duration in the CP group.

## Introduction

Atrial fibrillation (AF) has been associated with an increased risk for stroke, heart failure (HF), and all-cause mortality. Uncontrolled ventricular rates may lead to systolic dysfunction and HF. Atrioventricular node ablation (AVNA) with permanent pacemaker implantation is a highly effective treatment approach in AF patients with high ventricular rates resistant to other treatment modalities, especially in the elderly or those with severe comorbidities.^1^ AHA/ACC/HRS Atrial Fibrillation Practice Guidelines (2014) recommend that AVNA with permanent right ventricular pacing (RVP) is a reasonable strategy to control the heart rate in AF, when pharmacological therapy is inadequate and rhythm control cannot be achieved (class IIa, level of evidence B).^2^ Cardiac resynchronization therapy with biventricular pacing (BVP) should be considered in patients with reduced left ventricular ejection fraction (LVEF) who are candidates for AVNA for rate control (class IIA, level of evidence B).^2^ The APAF-CRT trial demonstrated a significant reduction in mortality in patients with a narrow intrinsic QRS duration and HF who were randomized to AVNA and BVP compared to pharmacological rate control.^3^

Conduction system pacing (CSP) using His bundle pacing (HBP) or left bundle branch area pacing (LBBAP) has developed as an alternative to conventional pacing (CP) using RVP or BVP.^4^^,^^5^ Previously, HBP and AVNA was shown to be feasible and associated with improved LVEF and functional class.^6^, ^7^, ^8^ Recently LBBAP and AVNA was shown to be feasible, safe, and associated with low capture thresholds.^7^^,^^9^^,^^10^ However, there are no studies comparing the clinical outcomes of CP vs CSP in this setting. The aim of this study is to report on the clinical outcomes of CSP compared with CP in patients undergoing AVNA, using real-world experience.

## Methods

### Study design

This was a retrospective observational study of patients undergoing AVNA in the Geisinger Health System between January 2015 and October 2020. The study included patients aged >18 years who underwent AVNA with device implantation owing to AF with uncontrolled ventricular rates refractory to pharmacological therapy and with >6 months of follow-up. Patients were excluded if they were <18 years of age or had <6 months of follow-up. The Institutional Review Board approved the study. All patients provided written informed consent prior to the procedure. The research reported in this paper adhered to Helsinki Declaration (as revised in 2013) guidelines. The data underlying this article will be shared on reasonable request to the corresponding author.

### Procedure

#### Conduction system pacing

HBP or LBBAP using a SelectSecure pacing lead (Medtronic, model 3830) and a delivery sheath (Medtronic, C315His or C304His SelectSite) was performed as previously reported.^4^^,^^11^, ^12^ Following successful implantation of the lead at the His bundle location or left bundle branch area, conduction system capture thresholds were documented. If HBP was performed, we preferentially chose a distal HBP site with atrial electrograms <0.5 mV based on our previous experience to avoid threshold increase during AVNA.^6^ In some patients, both HBP and LBBAP leads were implanted, wherein the LBBAP lead served as a back-up lead to HBP.

#### Conventional pacing

Right ventricle (RV) leads and coronary sinus leads were implanted in standard fashion. Left ventricle (LV) leads were placed preferentially at the mid to basal posterolateral LV region based on coronary venous anatomy, phrenic nerve stimulation, and qLV as appropriate.

#### Choice of pacing

The choice of CSP vs CP was based on the operator preference and individual hospital practice. Implanters at the Geisinger Wyoming Valley Medical Center exclusively performed CSP, while the operators at Geisinger Medical Center and Geisinger Community Medical Center preferentially performed CP. Within CP, the choice of RVP vs BVP was based on underlying LV dysfunction, history of HF, or associated comorbidities such as chronic renal failure and frailty. Prior to 2018 CSP was performed using HBP, while in the latter years LBBAP was preferentially performed if HBP had capture thresholds >1.5 V or a distal HBP site could not be achieved.

#### Atrioventricular node ablation

AVNA was preferentially performed at the time of device implantation in both CSP and CP. In short, following the device implantation, the compact atrioventricular (AV) nodal region with a far-field His electrogram and a large atrial electrogram was targeted. In patients undergoing HBP, ablation was targeted at the compact AV nodal region near the ring electrode. Care was taken to avoid ablating adjacent to the tip electrode.^6^ In a small number of patients, AVNA was performed several weeks or months after the device implantation.

#### Programming in CSP

CSP leads were connected to the right atrial, RV, or LV port in an individual patient, depending on the type of device implanted. In patients undergoing both HBP and LBBAP, timing between HBP and LBBAP was adjusted to allow LBBAP to fall in the refractory period. When the CSP lead was connected to the RV port in a dual-chamber device, AV delay was shortened to permit conduction through the His-Purkinje system prior to ventricular activation. When the CSP lead was connected to the atrial port, the device was programmed to DVI or DDI mode with AV delay of 100 ms to allow for ventricular sensing and inhibition. When the CSP lead was connected to the LV port, the device was programmed with maximal LV-RV delay (80–100 ms).

### Data collection and follow-up

Patient demographics, medical history, medications, heart failure hospitalizations (HFH), date of HFH, death, and date of death were collected using data query through the electronic medical records and verified by manual chart review. Electrocardiogram characteristics, echocardiographic parameters, implant information, and electrical characteristics of pacing leads were documented.

Patients were followed in device clinic at 2 weeks, at 3 months, and yearly thereafter. Additionally, patients were followed via remote device monitoring at every 3 monthly intervals in between scheduled office visits. Ventricular pacing burden was routinely documented in all patients. Any increase in pacing threshold by >1 V or need for lead revisions were documented. QRS duration at baseline and following pacing were documented. To ensure uniformity, paced QRS duration was measured from the first ventricular pacing spike to the end of QRS on a 12-lead electrocardiogram. Only in the case of selective HBP at programmed output was QRS duration measured from QRS onset to offset.

The primary outcome measured was the combined endpoint of first episode of HFH or death from any cause. Secondary outcomes included individual outcomes of death from any cause and HFH, and subgroup analysis of the primary combined endpoint of death or HFH in patients with LVEF <50% and LVEF ≥50%. HFH was defined as an unplanned outpatient or emergency department visit or inpatient hospitalization in which the patient presented with signs and symptoms consistent with HF and required intravenous diuretic therapy. Safety endpoints included threshold increase >1 V and need for lead revisions.

### Statistics

All data were summarized using frequencies and percentages for categorical data and mean (± standard deviation) for continuous data (distribution dependent). Descriptive statistics were reported for the full sample and stratified by CP and CSP groups. Comparisons between groups were made with *t* test or χ^2^ analysis. Two-sided *P* value of <.05 was considered significant. Univariate and multivariate Cox proportional hazard models were used to estimate survival probability for composite primary outcome and secondary outcomes of mortality and HFH by CP and CSP groups. Initially, univariate analysis was carried out using variables previously determined to be clinically significant. Univariate predictors with *P* value <.10 were entered into multivariate Cox proportional hazard models to determine significant independent predictors. Patients’ last follow-up dates were determined by the last time they were seen in the Geisinger Health System, or until the time of death, whichever occurred first. All data and follow-up dates were censored after April 30, 2021. For Cox survival analyses, time censoring was determined by time to event (primary or secondary) or time to last follow-up in the Geisinger Health System, whichever came first. All analysis was performed using SPSS Statistics version 27.0 (SPSS, Chicago, IL).

## Results

### Baseline characteristics

A total of 223 patients underwent AVNA at Geisinger Health System during the study period (110 with CSP and 113 with CP). Patients were followed for a mean duration of 27 ± 19 months. Table 1 shows the baseline demographics of the study population: mean age 75 ± 10 years; male 52%; hypertension 67%; coronary artery disease 40%; LVEF 43% ± 15%, and baseline QRS duration of 111 ± 30 ms. While the baseline demographics, distribution of various types of AF, and medication use were similar between the 2 groups, LVEF was lower, QRS duration was wider, and there was a higher number of patients with left bundle branch block in the CP group (*P* < .01). Baseline characteristics of the study group with LVEF <50% and those with LVEF ≥50% are shown in Table 2. In the subgroup with LVEF <50%, QRS duration was wider (130 ± 31 ms vs 109 ± 24 ms, *P* < .01) and LVEF lower (28% ± 10% vs 35% ± 9%, *P* < .01) in the CP group compared to CSP. No significant differences were noted among the patients with LVEF ≥50%.

**Table 1:** Baseline characteristics of the study population

	All patients (n = 223)	CSP patients (n = 110)	CP patients (n = 113)	*P* value
Age (y)	75 ± 10	75 ± 10	75 ± 10	1
BMI	29 ± 7	29 ± 7	30 ± 6	.53
Male sex	115 (52%)	61 (55%)	54 (48%)	.25
CAD	89 (40%)	47 (45%)	42 (37%)	.7
DM	55 (25%)	31 (28%)	24 (21%)	.71
HTN	150 (67%)	85 (77%)	65 (57%)	.24
AF type				.55
Paroxysmal	48 (21%)	21 (19%)	27 (24%)	
Persistent	134 (60%)	70 (63%)	64 (57%)	
Permanent	41 (18%)	19 (17%)	22 (20%)	
Baseline EF (%)	43 ± 15	47 ± 14	39 ± 16	<.01
EF <50%	114 (51%)	47 (43%)	67 (60%)	.01
Baseline QRS duration (ms)	111 ± 30	103 ± 25	119 ± 32	<.01
Baseline QRS morphology				
Normal QRS	123 (56%)	74 (68%)	49 (43%)	<.01
RBBB	25 (11%)	12 (11%)	13 (12%)	.88
LBBB	48 (22%)	11 (10%)	37 (32%)	<.01
IVCD	24 (11%)	12 (11%)	12 (11%)	.94
Medications				
Beta blockers	200 (90%)	103 (94%)	97 (86%)	.06
Calcium channel blockers	35 (16%)	15 (14%)	20 (18%)	.4
Digoxin	55 (25%)	26 (24%)	29 (26%)	.6
Antiarrhythmic agents	147 (66%)	76 (69%)	71 (63%)	.32
ARB/ACEI/ARNI	122 (55%)	56 (51%)	66 (58%)	.29

AF = atrial fibrillation; ARB = angiotensin receptor blocker; ACEI = angiotensin-converting enzyme inhibitor; ARNI = angiotensin receptor neprilysin inhibitor; BMI = body mass index; CAD = coronary artery disease; CP = conventional pacing; CSP = conduction system pacing; DM = diabetes mellitus; EF = ejection fraction; HTN = hypertension; IVCD = intraventricular conduction delay; LBBB = left bundle branch block; RBBB = right bundle branch block.

**Table 2:** Patient characteristics based on baseline left ventricular ejection fraction

	LVEF ≥50% (n=109)			LVEF <50% (n=114)		
	CSP (n = 63)	CP (n = 46)	*P* value	CSP (n = 47)	CP (n = 67)	*P* value
Type of pacing	HBP 51 (81%) LBBAP 12(19%)	RVP 40 (87%) BVP 6 (13%)		HBP 33(70%) LBBAP 14 (30%)	BVP 51 (76%) RVP 16 (24%)	
Age (y)	76 ± 10	78 ± 8	.13	73 ± 10	72 ± 10	.50
BMI	29.7 ± 8	28.7 ± 6.2	.51	28 ± 6	30 ± 7	.08
Male sex	46 (73%)	32 (67%)	.69	32 (68%)	45 (67%)	.91
CAD	26 (41%)	15 (32%)	.35	22 (47%)	30 (45%)	.89
DM	19 (30%)	10 (22%)	.32	12 (26%)	17 (26%)	.94
HTN	50 (79%)	31 (67%)	.16	36 (77%)	50 (76%)	.92
AF type			.74			.55
Paroxysmal	15 (24%)	14 (30%)		6 (13%)	13 (19%)	
Persistent	39 (62%)	26 (57%)		31 (66%)	38 (57%)	
Permanent	9 (14%)	6 (13%)		10 (21%)	16 (24%)	
Baseline EF (%)	57 ± 4	56 ± 5.2	.30	35 ± 9	28 ± 10	<.01
Baseline QRS duration (ms)	97 ± 24	103 ± 26	.23	109 ± 24	130 ± 31	<.01
Baseline QRS morphology						
Normal QRS	49 (78%)	30 (65%)	.15	25 (53%)	19 (28%)	<.01
RBBB	9 (14%)	4 (9%)	.37	3 (6%)	9 (13%)	.22
LBBB	3 (5%)	5 (11%)	.23	8 (17%)	32 (48%)	<.01
IVCD	1 (2%)	6 (13%)	.02	11 (23%)	6 (9%)	.03
Medications						
Beta blockers	59 (94%)	35 (76%)	<.01	44 (94%)	62 (93%)	.82
Calcium channel blockers	9 (14%)	13 (28%)	.07	6 (13%)	7 (11%)	.70
Digoxin	13 (21%)	6 (13%)	.3	13 (28%)	23 (34%)	.39
Antiarrhythmic agents	46 (73%)	33 (71%)	.88	30 (64%)	38 (57%)	.45
ARB/ACEI/ARNI	30 (48%)	18 (39%)	.38	26 (57%)	48 (71%)	.29

BVP = biventricular pacing; HBP = His bundle pacing; LBBAP = left bundle branch area pacing; RVP = right ventricular pacing; other abbreviations as in Table 1.

### Procedural characteristics

Procedure times were longer in the CSP group compared to the CP group (130 ± 67 minutes vs 101 ± 65 minutes, *P* < .01) but the fluoroscopy times were comparable (17.4 ± 12 minutes vs 15.6 ± 16 minutes, *P* = .39). In the CSP group, 84 patients underwent HBP and in 46 patients LBBAP leads were implanted. In 20 of these patients, both HBP and LBBAP leads were implanted, with the LBBAP lead serving as the back-up pacing lead. RV back-up pacing leads were also implanted in 34 of the CSP patients. Of the 84 patients with HBP, the lead was connected to the atrial port in 26, RV port in 28, and LV port in 30 patients. Among the 46 patients with LBBAP, the lead was connected to the atrial port in 3, RV port in 34, and LV port in 9 patients. In the CP group, traditional BVP with RV and coronary sinus leads were implanted in 57 patients and RV-only pacing was performed in 56 patients. A higher number of patients in the CP group had implantable cardioverter-defibrillator and BVP devices compared to the CSP group (*P* < .01). In both groups >85% of patients underwent simultaneous AVNA and device implantation (87% in CP vs 94% in CSP group). In the remainder, most patients underwent ablation within 2 months after device implantation.

**Figure 1:**
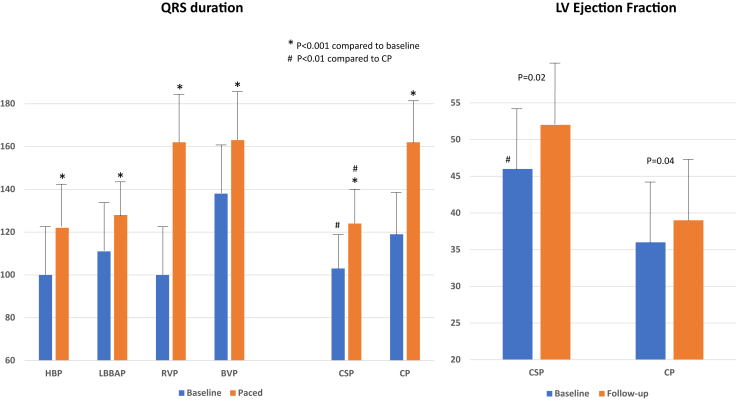
Electrocardiogram and left ventricular (LV) function change post atrioventricular node ablation: QRS duration and LV ejection fraction at baseline and during various modalities of pacing are shown. BVP = biventricular pacing; CP = conventional pacing; CSP = conduction system pacing; HBP = His bundle pacing; LBBAP = left bundle branch area pacing; RVP = right ventricular pacing.

Procedural, electrical, and echocardiographic characteristics of the study groups are shown in Table 3. In the CSP group, QRS duration increased from 103 ± 25 ms at baseline to 124 ± 20 ms during pacing (*P* < .01) compared to 119 ± 32 ms to 162 ± 24 ms in the CP group (*P* < .001). Paced QRS duration was significantly narrower in CSP compared to CP (*P* < .001) (Figure 1). There were no significant differences in the paced QRS duration within groups (HBP vs LBBAP and RVP vs BVP).

**Table 3:** Procedural, electrical, and echocardiographic outcomes

	Conduction system pacing (n = 110)		Conventional pacing (n = 113)		*P* value
Procedure duration (min)	130 ± 67		101 ± 65		<.01
Fluoroscopy duration (min)	17 ± 12		16 ± 15		.39
Device type					
Pacemaker	91 (83%)		65 (58%)		<.01
ICD	19 (17%)		48 (42%)		<.01
Single-chamber	9 (8%)		21 (19%)		<.01
Dual-chamber	62 (56%)		34 (30%)		<.01
Biventricular	39 (36%)		58 (51%)		<.01
Electrical characteristics	HBP	LBBAP	RVP	BVP (CS)	
Ventricular pacing leads, n (%)	84 (76%)	46 (42%)	56 (50%)	57 (50%)	
Baseline QRSd (ms)	100 ± 23	111 ± 29	100 ± 21	138 ± 28	<.01
Paced QRSd (ms)	122 ± 21	128 ± 14	162 ± 21	163 ± 26	<.01
Pacing threshold (implant)	1.11 ± 0.7	0.63 ± 0.29	0.7 ± 0.33	1.38 ± 1.34	
Pacing threshold (last f/u)	1.1 ± 0.6	0.73 ± 0.22	0.55 ± 0.43	1.2 ± 0.74	
Threshold increase >1 V	6	0	0	3	
Lead revision/abandoned	2	0	5	1	
LVEF	CSP (n = 77)		CP (n = 83)		
Baseline LVEF	46.5 ± 14.2		36.4 ± 16.1		<.01
Follow-up LVEF	51.9 ± 11.2∗		39.5 ± 16.0∗		<.01
Subgroups	HBP (58)	LBBAP (19)	RVP (41)	BVP (42)	
Baseline LVEF	46.4 ± 14.1	46.9 ± 15.1	50.3 ± 12.0	26.7 ± 10.5	
Follow-up LVEF	52.3 ± 10.9∗	50.1 ± 12.9	47.7 ± 13.2	33.8 ± 15.5∗	

CS = coronary sinus; f/u = follow-up; ICD = implantable cardioverter-defibrillator; other abbreviations as in Tables 1 and 2.

∗ *P* < .05 compared to baseline.

Pacing thresholds remained relatively stable in both groups as a whole and in the subgroups. Capture thresholds in the HBP and coronary sinus leads were higher than those obtained in the LBBAP and RVP group at baseline and during last follow-up (*P* < .01). In the HBP group, 6 (7.2%) patients had >1 V increase in capture thresholds during follow-up: the HBP lead was removed with simple traction and replaced with LBBAP in 1 patient at 32 months owing to early battery depletion and HBP was turned off in another patient (18 months) in whom LBBAP was used. No threshold increase or lead revisions were required in the LBBAP group. In the RVP group, 3 patients required lead revisions and upgrade to BVP was required in 2 other patients. Threshold increase >1 V was observed in 3 patients with coronary sinus lead.

Infections requiring system removal were observed in 3 patients (1 each in RVP, LBBAP, and BVP). AVNA was unsuccessful from right-sided approach in 1 patient. Repeat AVNA was performed owing to conduction recovery in 6 (2.5%) patients (HBP 1, LBBAP 3, BVP 2). In another patient with HBP, AV conduction partially recovered but did not require repeat ablation owing to controlled ventricular rates. Acute renal failure requiring temporary dialysis occurred in 1 patient (LBBAP) with preexisting chronic kidney disease and diabetes.

### Echocardiographic data

Follow-up echocardiograms were available in 160 (72%) patients. In the CSP group (n = 77), LVEF improved from 46.5% ± 14.2% at baseline to 51.9% ± 11.2% during follow-up (*P* = .02). Similarly, LVEF improved from 36.4% ± 16.1% at baseline to 39.5% ± 16.0% during follow-up (*P* = .04) in the CP group (n = 83). In both the HBP and BVP subgroups, LVEF increased significantly, from 46.4% ± 14.1% to 52.3% ± 10.9% (*P* < .001) and from 26.7% ± 10.5% to 33.8% ± 15.5% (*P* < .001), respectively. However, there was a trend toward nonsignificant decrease in LVEF in patients with RVP (50.3% ± 12.0% to 47.7% ± 13.2%, *P* = .2). There was a trend toward nonsignificant increase in LVEF in patients with LBBAP (46.9% ± 15.1% to 50.1% ± 12.9%, *P* = .2).

### Clinical outcomes

**Figure 2:**
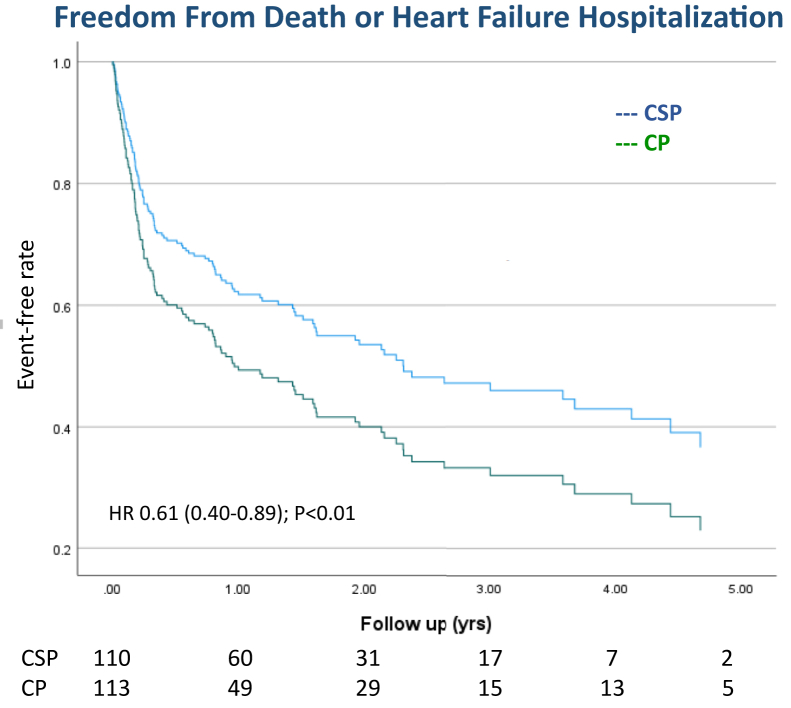
Primary composite outcome of time to death or heart failure hospitalization among patients undergoing atrioventricular node ablation and conduction system pacing (CSP) vs conventional pacing (CP). Survival curves and analysis show a reduction in the primary composite outcome (all-cause mortality or heart failure hospitalization) associated with CSP when compared to CP.

**Figure 3:**
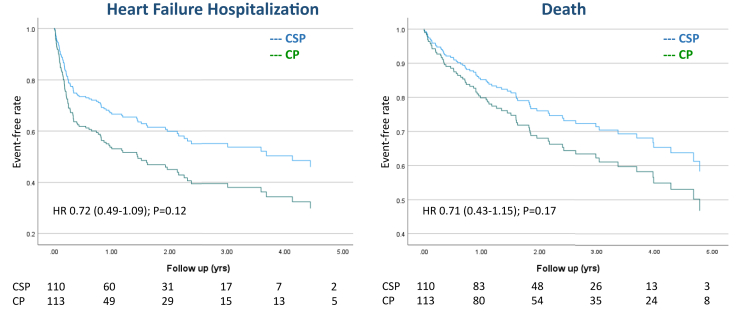
Individual secondary outcomes of time to death and heart failure hospitalizations among patients undergoing atrioventricular node ablation and conduction system pacing (CSP) vs conventional pacing (CP). Survival curves and analysis demonstrate no significant differences in the secondary outcomes of heart failure hospitalization and all-cause mortality.

The primary outcome (combined endpoint of death from any cause or HFH) occurred in 48% of patients in the CSP group vs 62% of patients in the CP group (hazard ratio [HR] 0.61, 95% confidence interval [CI] 0.42–0.89, *P* < .01) (Figure 2). On multivariate analysis, history of HF and no beta-blocker therapy were also predictive of worse outcomes (Table 4). During the study period, there was a nonsignificant trend toward fewer deaths (secondary endpoint) in the CSP group compared to the CP group (25% vs 37%; HR 0.71, 95% CI 0.43–1.15, *P* = .17). The incidence of individual secondary endpoint of HFH decreased in the CSP group compared to CP (39% vs 50%; HR 0.72, 95% CI 0.49–1.09, *P* = .12) but did not reach statistical significance (Figure 3, Table 5). In subgroup analysis of patients undergoing CSP, there was no significant difference in the primary outcome of death or HFH between HBP and LBBAP (HR 0.75, 95% CI 0.38–1.5, *P* = .41) (Supplemental Figure 1). Similarly, there was no significant difference in the primary outcome when RVP was compared to BVP (HR 0.72, 95% CI 0.45–1.1, *P* = .18).

**Table 4:** Univariate and multivariate hazard ratio for composite outcome of time to all-cause death or heart failure hospitalization

Parameters	Univariate analysis			Multivariate analysis I			Multivariate analysis II		
	HR	95% CI	*P* value	HR	95% CI	*P* value	HR	95% CI	*P* value
CSP vs CP	0.68	0.48–0.98	.04	0.53	0.31–0.91	.02	0.61	0.42–0.89	<.01
Age	1.01	0.99–1.0.3	.155						
Female	0.82	0.58–1.2	.27						
Hypertension	1.7	1.1–2.5	.03	1.56	0.94–2.5	.07			
Diabetes	1.64	1.12–2.4	.01	1.5	0.98–2.30	.06			
CAD	1.47	1.0–2.1	.03	1.3	0.89–1.9	.17			
History of CHF	1.8	1.3–2.7	<.01	1.9	1.1–3.2	<.01	1.85	1.3–2.7	<.01
Type of AF	0.48	0.29–0.78	<.01	0.62	0.34–1.1	.06			
Beta blockers	0.36	0.24–0.95	.02	0.42	0.19–0.98	.05	0.39	0.18–0.86	.02
ACE/ARB/ARNI	1.27	0.88–1.8	.2						
Baseline QRS	1	1–1.1	.02	1.0	0.99–1.1	.99			
Paced QRS	1.1	0.99–1.0	.08	1.0	0.99–1.1	.82			
Baseline LVEF	0.99	0.98–1.01	.07	1.0	0.98–1.03	.57			
EF <50%	1.4	0.95–1.95	.08	0.99	0.46–2.1	.98			
CSP vs BVP	0.59	0.39–0.89	.01	0.6	0.32–1.3	.21			
HBP vs LBBAP	0.80	0.41–1.58	.53						
CSP vs RVP	0.85	0.56–1.33	.47						
BVP vs RVP	0.72	0.45–1.1	.19						

CHF = congestive heart failure; other abbreviations as in Tables 1 and 2.

**Table 5:** Clinical outcomes (univariate and multivariate analysis using the clinical variables in Table 4)

Clinical outcomes	Total	CSP	CP	*P* value	Univariate			Multivariate		
					HR	95% CI	*P* value	HR	95% CI	*P* value
Death or HFH, n (%)	123 (55%)	53 (48%)	70 (62%)	.04	0.68	0.48–0.98	.04	0.61	0.42–0.89	<.01
Mortality, n (%)	69 (31%)	27 (25%)	42 (37%)	.04	0.71	0.43–1.15	.168			
HFH, n (%)	99 (44%)	43 (39%)	56 (50%)	.03	0.64	0.42–0.95	.03	0.72	0.49–1.09	.12

CP = conventional pacing; CSP = conduction system pacing; HFH = heart failure hospitalization.

**Figure 4:**
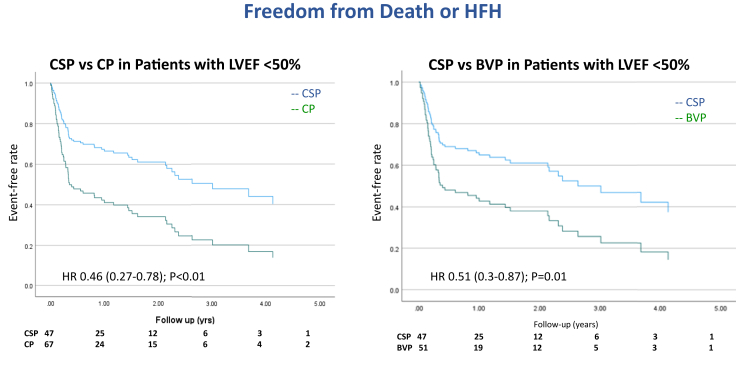
Subgroup analysis of primary composite outcome of time to death or heart failure hospitalizations among patients with left ventricular ejection fraction (LVEF) <50%. Conduction system pacing (CSP) vs conventional pacing (CP) and CSP vs biventricular pacing (BVP). Cox regression survival curves and multivariate analysis demonstrate significant reduction in the primary composite outcome (all-cause mortality or heart failure hospitalization) among patients with LVEF <50% when CSP was compared with CP or BVP.

In a subgroup analysis of patients with LVEF <50% at baseline (n = 114), the incidence of death or HFH was reduced in patients with CSP compared to CP (HR 0.46, 95% CI 0.27–0.78, *P* < .01). Similarly, when CSP was compared with only BVP in patients with LVEF <50% (n = 98), the incidence of death or HFH was reduced (HR 0.51, 95% CI 0.3–0.87, *P* = .01) (Figure 4). Among patients with LVEF ≥50% (n = 109), there was no significant difference between CSP and CP in the combined endpoint of death or HFH (HR = 1.1, 95% CI 0.64–1.9, *P* = .71; Supplemental Figure 1).

## Discussion

In this real-world, nonrandomized, retrospective, observational study, we investigated the clinical outcomes of CSP using HBP or LBBAP compared to CP with RVP or BVP in patients undergoing AVNA for AF refractory to medical therapy. Our major findings are the following: (1) CSP was feasible and safe in patients undergoing AVNA with similar electrical and procedural outcomes when compared to CP; and (2) CSP was associated with a reduction in the primary outcome of death or HFH compared to CP, although this was likely owing to a sicker group of patients with lower baseline LVEF and wider QRS duration in the CP group.

Case series and randomized trials of AVNA with permanent pacemaker implantation have proven to be an effective therapeutic option for improving symptoms and quality of life in AF patients with high ventricular rates resistant to other treatment modalities.^13^, ^14^, ^15^ AHA/ACC/HRS Atrial Fibrillation Practice Guidelines (2014) recommend that AVNA with permanent RVP is a reasonable strategy to control the heart rate in AF, when pharmacological therapy is inadequate and rhythm control cannot be achieved (class IIa, level of evidence B).^2^ Brignole and colleagues^14^ reported clinical outcomes on 186 patients with permanent AF who underwent AVNA and were then randomized to receive either BVP or RVP. The primary composite endpoint of death from HF, HFH, and worsened HF was reduced by 63% in BVP compared to RVP during a median follow-up of 20 months. Similarly, Doshi and colleagues^13^ reported improvement in quality of life, 6-minute walk distance, and ejection fraction in 184 patients undergoing AVNA and BVP compared to RVP, especially in patients with baseline LVEF <45% and those with class II/III symptoms. In a retrospective, observational study based on insurance claims of 1611 patients with AV junction ablation (798 RVP and 363 BVP), while AF hospitalizations were reduced in both groups, BVP was associated with 38% reduced risk of HFH compared to RVP.^16^ In a large study of 1810 patients with AV block (mean age 73.5 years) randomized to either RVP (n = 908) or BVP (n = 902), after a mean follow-up of 5.6 years, the groups had a similar rate of the composite endpoint of time to death or first HFH (HR 0.87, *P* = .08).^17^ While effective rate control achieved by AVNA significantly improves symptoms, the long-term deleterious effects of RVP in patients with reduced ejection fraction has led to a search for alternative pacing modalities.

Recently, HBP and LBBAP has been shown to be feasible and effective in maintaining physiologic ventricular activation and improving clinical outcomes following AVNA in small observational studies.^6^, ^7^, ^8^, ^9^, ^10^ These studies demonstrated that AVNA and CSP with HBP or LBBAP is associated with improvement in LVEF and functional class. AVNA can be challenging in some patients with HBP owing to risk for increase in HBP threshold when ablating in close proximity to the pacing lead.^6^ However, in these patients LBBAP is an attractive alternative to achieve low and stable pacing thresholds.^7^ In our study, both HBP and LBBAP resulted in significantly narrower QRS duration than CP, suggesting more physiologic ventricular activation with CSP.

In this observational real-world study, we compared the procedural and clinical outcomes of CSP vs CP using RVP or BVP in a large group of patients. While the procedural duration was slightly longer in the CSP group, overall capture thresholds were comparable to CP and remained relatively stable during a follow-up of 27 ± 19 months. Threshold increase >1 V was seen in 7.2% of patients with HBP compared to 5.2% of patients with BVP. More patients in the RVP group (9%) required lead revisions owing to perforation, lead dislodgement, or upgrade to BVP compared to the HBP group (2.3%). Based on these observations, CSP appears to be a safe and acceptable alternative approach in patients undergoing AVNA compared to CP. However, there was a slightly higher incidence of recurrence of AV node conduction in the CSP group compared to the CP group (5 vs 2), likely owing to reluctance in delivering longer or additional ablation lesions for fear of increasing HBP thresholds.

As seen in randomized studies of BVP vs RVP and AVNA, there was significant improvement in LVEF in patients undergoing BVP and HBP while there was a trend toward reduction in LVEF in the RVP group. This study provides support to the value of maintaining ventricular synchrony, in addition to achieving adequate rate control with AVNA, by using CSP or BVP.

In a randomized study of 133 patients, Brignole and colleagues^3^ demonstrated a significant reduction in the primary endpoint of mortality in those undergoing AVNA and BVP compared to medical therapy, during a median follow-up of 29 months (11% vs 29%; HR 0.26, CI 0.10–0.65, *P* = .004). The combined secondary endpoint of death or HFH was also significantly reduced with BVP and AVNA (29% vs 51%; HR 0.40, 95% CI 0.22–0.73, *P* = .002). This benefit was seen irrespective of whether baseline LVEF was <35% or >35%. In our study, the combined endpoint of death or HFH was significantly reduced (48% vs 62%; HR 0.61, 95% CI 0.42–0.89, *P* < .01) in patients with CSP compared to CP. Also, there was a trend toward reduction in the secondary endpoints of individual outcomes of death and HFH without reaching statistical significance. However, this difference may have been due to sicker patients with lower LVEF and wider QRS duration in the CP group in this nonrandomized study. It is also likely that undertreatment of HF in patients with low ejection fraction may have impacted the outcomes. Among patients with LVEF ≥50%, there were no significant differences in the primary outcome between the 2 groups.

Our study supports the hypothesis that AVNA and CSP has the potential to achieve excellent rate control without the risks of ventricular dyssynchrony induced by ventricular pacing, possibly translating into improved clinical outcomes.

### Limitations

This was a real-world, nonrandomized, on-treatment analysis of patients undergoing AVNA in a large health system. The type of pacing (CP vs CSP) was determined by clinical practice at each institution and their expertise. While the baseline characteristics of the study population were similar in both groups, significant differences existed in terms of baseline LVEF and QRS duration. Nonetheless, these factors were not significant on multivariate analysis. Additionally, echocardiographic data were not available in all patients during follow-up. Given the lack of homogeneity among the study population, the results should be interpreted with caution and should be considered as hypothesis-generating rather than proof. Large, randomized clinical trials comparing CSP vs BVP in patients undergoing AVNA are necessary to confirm clinical benefits.

## Conclusion

CSP appears to be a safe and effective option for pacing in patients with AF undergoing AVNA in a high-volume CSP center.
