# Supplementary material for: Conduction system pacing versus conventional pacing in patients undergoing atrioventricular node ablation: Nonrandomized, on-treatment comparison
Source: Heart Rhythm O2. 2022 May 4;3(4):368–76. doi: 10.1016/j.hroo.2022.04.005 (PMC9463688; doi:10.1016/j.hroo.2022.04.005)
Supplement: Supplementary Figure 1 [file mmc1.pptx]

## Slide 1
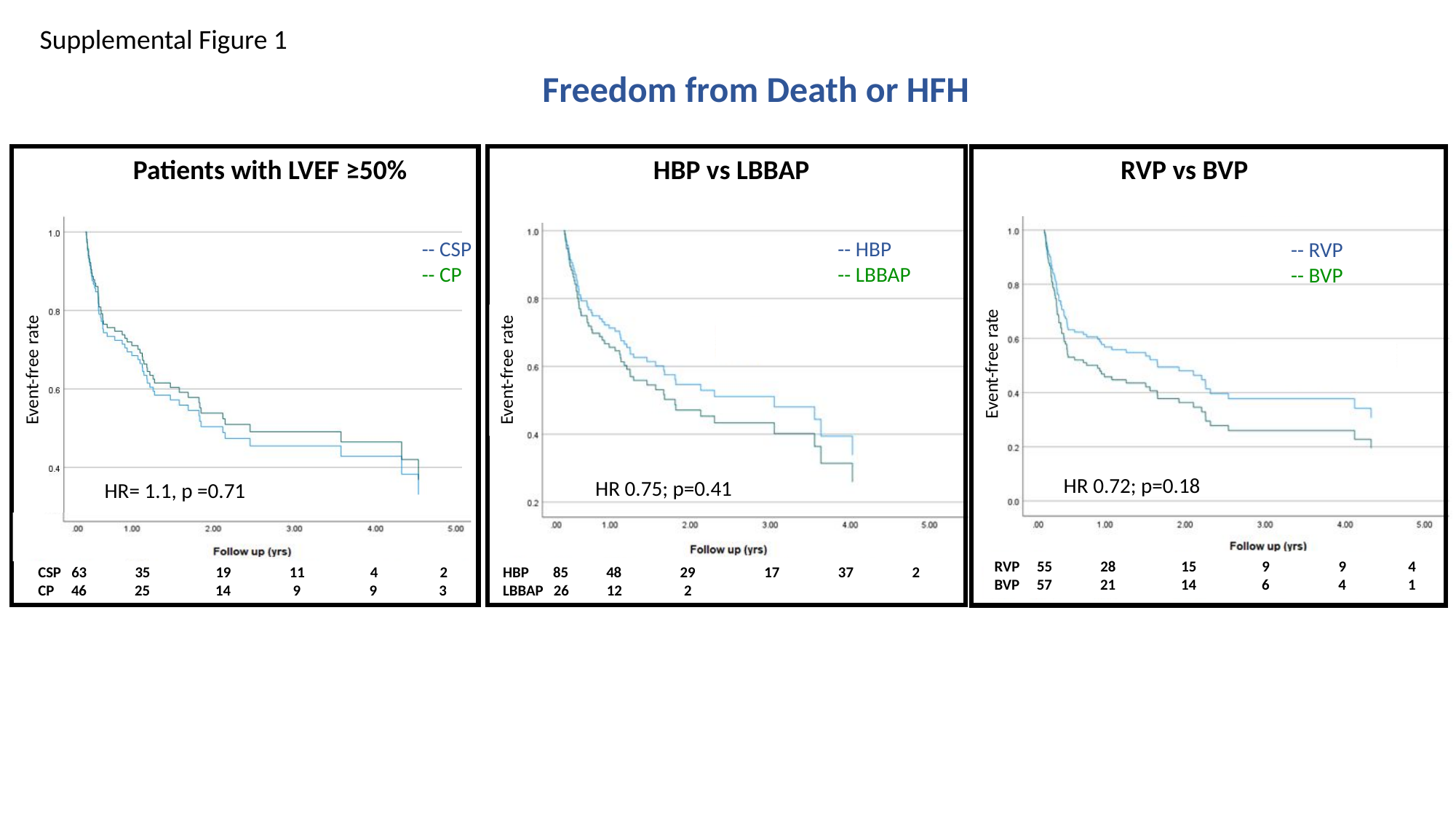

Supplemental Figure 1
Freedom from Death or HFH
Patients with LVEF ≥50%
HBP vs LBBAP
RVP vs BVP
-- CSP
-- CP
-- HBP
-- LBBAP
-- RVP
-- BVP
HR 0.72; p=0.18
HR 0.75; p=0.41
RVP 55 28 15 9 9 4
BVP 57 21 14 6 4 1
CSP 63 35 19 11 4 2
CP 46 25 14 9 9 3
HBP 85 48 29 17 37 2
LBBAP 26 12 2
HR= 1.1, p =0.71
Event-free rate
Event-free rate
Event-free rate

## Slide 2
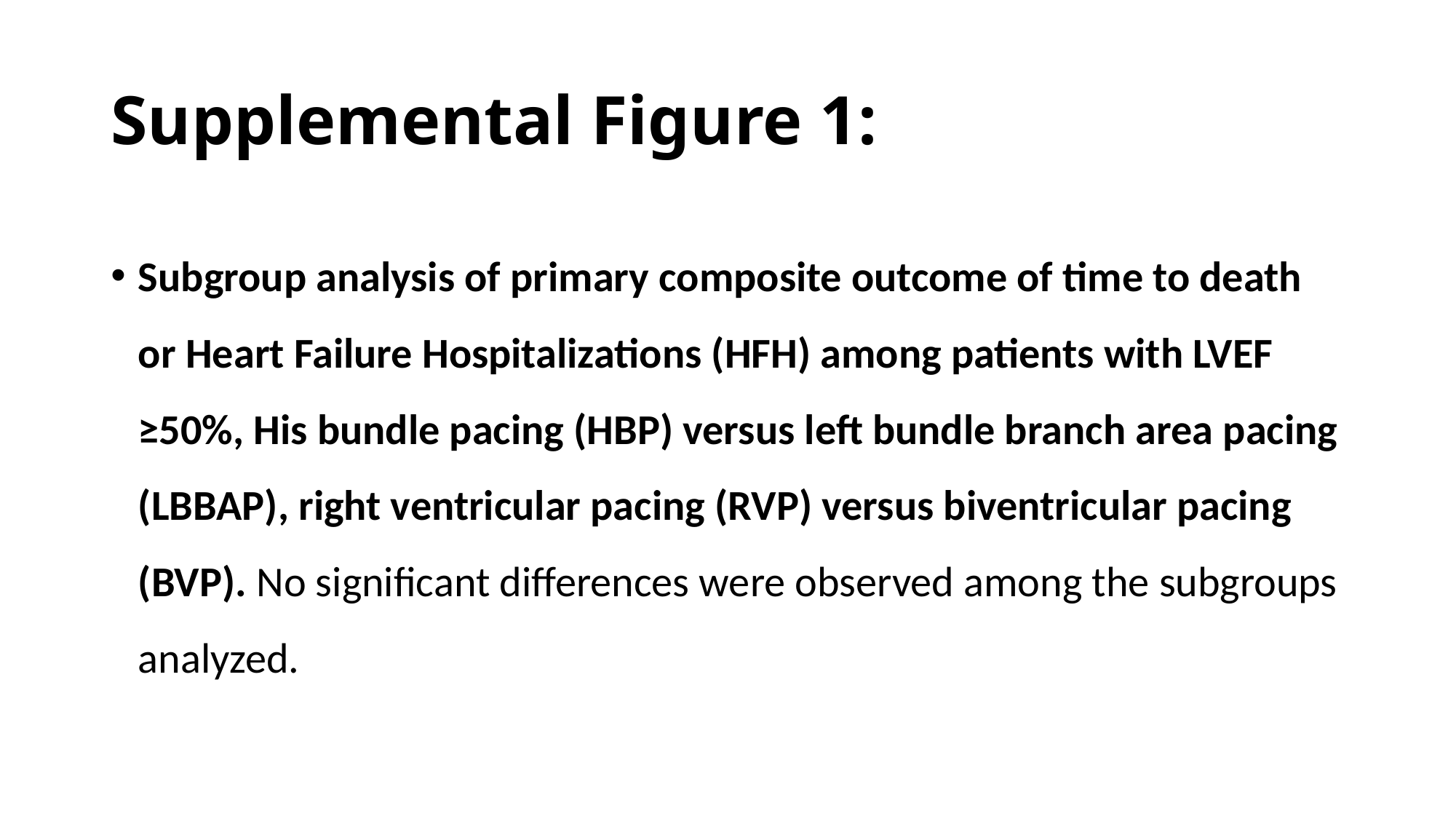

# Supplemental Figure 1:
Subgroup analysis of primary composite outcome of time to death or Heart Failure Hospitalizations (HFH) among patients with LVEF ≥50%, His bundle pacing (HBP) versus left bundle branch area pacing (LBBAP), right ventricular pacing (RVP) versus biventricular pacing (BVP). No significant differences were observed among the subgroups analyzed.
